# The prognosis of critically ill patients with invasive group A streptococcus infection

**DOI:** 10.1186/s13054-020-03167-z

**Published:** 2020-07-14

**Authors:** Toshihide Izumida, Teruhiko Imamura

**Affiliations:** 1 Division of Community Medicine, Kanazawa Medical University Himi Municipal Hospital, 1130 Kurakawa, Himi, Toyama Japan; 2 Second Department of Medicine, University of Toyama, 2630 Sugitani, Toyama, 930-0194 Japan

**Keywords:** Cardiology, Critical care medicine, Sepsis

To the Editor:

We read with great interest the article of Björck et al., demonstrating that critically ill patients with invasive group A streptococcus (iGAS) infection had a lower mortality risk compared to critically ill sepsis patients with other microorganisms. Furthermore, GAS classified with *emm1*/T1 was associated with lower mortality than GAS with non-*emm1*/T1 [1]. We have two concerns that should improve their findings.

The first concern is whether iGAS infection per se is associated with a lower risk of mortality compared with other microorganism infections. There might be several confounding factors in their cohort. The control group included many patients with hospital-acquired infections and commodities, such as malignancy and immunosuppression. The authors might need to adjust for these factors or exclude the patients with hospital-acquired infection to evaluate the impact of iGAS per se.

The second concern is the management of critically ill patients with iGAS infection. The mortality and morbidity of patients with iGAS in their cohort were lower than previously reported cohort [2]. Although GAS with *emm1*/T1 is a leading cause for necrotizing soft-tissue infection, the early diagnosis and prompt aggressive treatment for necrotizing fasciitis are the only way to improve mortality [3, 4]. The authors might need to describe their detailed management, including data of the time from presentation to the first debridement, to evaluate the effect of therapy on the mortality and morbidity of iGAS in the real-world setting.

## Data Availability

Not applicable.
